# Recurrent Malignant Struma Ovarii With Peritoneal Implants Following Prior Thyroid Carcinoma Treatment

**DOI:** 10.7759/cureus.89606

**Published:** 2025-08-08

**Authors:** Fiorella Apuy Rodríguez, María Luisa Alvarado Mora, María Laura Alvarado Fernández, María Jesús Arias Alvarado

**Affiliations:** 1 Faculty of Medicine, University of Costa Rica, San José, CRI

**Keywords:** malignant struma ovarii, papillary thyroid carcinoma, peritoneal implants, recurrent ovarian neoplasm, struma ovarii

## Abstract

Struma ovarii (SO) is a rare form of ovarian teratoma predominantly composed of thyroid tissue. While most cases follow a benign course, some may exhibit malignant transformation or extra-ovarian spread. We present the case of a 43-year-old woman with a history of SO previously treated with right oophorectomy and systemic chemotherapy, along with a separate diagnosis of papillary thyroid carcinoma managed with total thyroidectomy and radioactive iodine ablation. She remained asymptomatic for several years until routine surveillance imaging revealed stable pelvic lesions. Surgical exploration via exploratory laparotomy and suboptimal cytoreduction was performed, leading to the resection of multiple peritoneal implants. Histopathology confirmed recurrence of malignant SO. Postoperative recovery was uneventful, and systemic therapy was deferred due to limited supporting evidence. This case highlights the importance of clinical suspicion, surveillance, and individualized surgical management for recurrent SO.

## Introduction

Struma ovarii (SO) is an uncommon form of ovarian teratoma classified as a monodermal variant [1]. A monodermal teratoma refers to an ovarian tumor that consists mainly or entirely of one specific tissue type. SO is characterized by the presence of thyroid tissue constituting more than 50% of the tumor [2]. It accounts for approximately 1% of all ovarian tumors and represents 2-5% of ovarian teratomas, with only 5-10% of cases presenting malignant transformation. Its usual presentation is incidental or with nonspecific symptoms, such as abdominal discomfort, pelvic pain, or adnexal masses found on imaging [1,2]. In some rare cases, it causes clinical hyperthyroidism due to functional thyroid tissue, which complicates the diagnosis [3].

In most patients, preoperative suspicion begins from ultrasound or CT imaging, which may reveal a complex ovarian mass with solid cystic components, sometimes with colloid material that appears as high attenuation areas [4]. As malignant transformation is not usual, SO is clinically relevant as it may mimic ovarian cancer and requires both tailored surgical and oncological management [5].

## Case presentation

A 43-year-old woman with no significant past medical history presented with a prior diagnosis of SO, which had been managed with surgical excision, total abdominal hysterectomy, and right oophorectomy. She also had a history of papillary thyroid carcinoma previously treated with total thyroidectomy followed by radioactive iodine (RAI) ablation, achieving favorable clinical and biochemical responses.

Although the patient remained completely asymptomatic, she underwent routine surveillance with CT scans every four months. One year after the initial surgical resection, surveillance imaging revealed suspicious pelvic lesions. She was subsequently treated with systemic chemotherapy using a platinum-taxane regimen. A follow-up CT scan showed no evidence of abdominal lesions.

However, four months later, a new pelvic lesion was detected. Throughout this period, the patient remained asymptomatic, reporting no abdominal pain, gastrointestinal symptoms, or constitutional signs. Laboratory evaluation revealed normal thyroid function and undetectable thyroglobulin levels. CT imaging identified a 2 cm pelvic mass in the right lower quadrant, with no evidence of distant metastasis.

Given her oncologic history and imaging findings, the case was discussed with the institutional peritoneal carcinomatosis team, and surgical exploration was recommended. Serial CT scans showed a stable pelvic mass and implants in the right lower quadrant, with no signs of distant metastatic disease.

**Figure 1:**
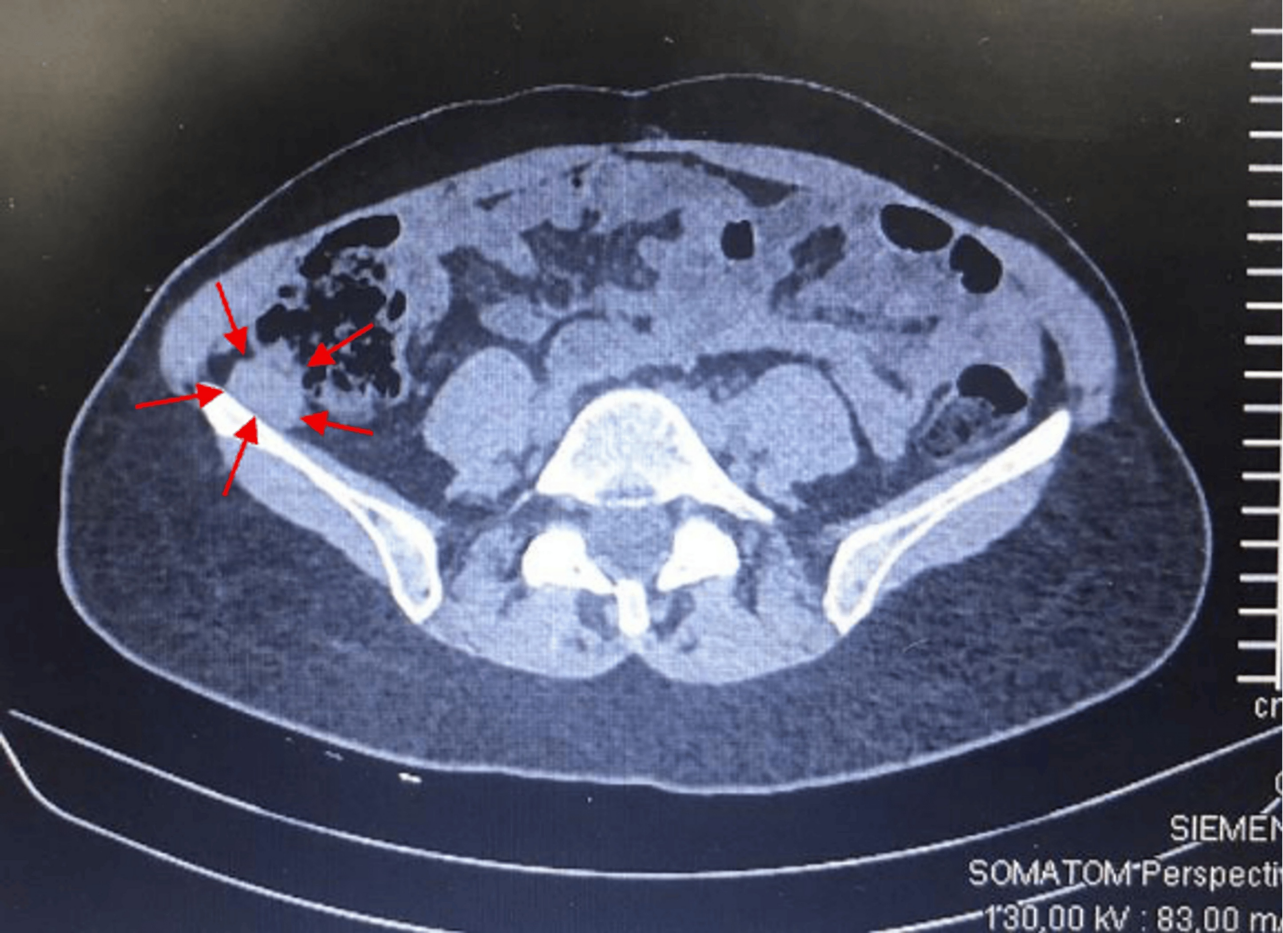
Abdominopelvic CT scan showing a space-occupying soft tissue density lesion in the right iliac fossa, with well-defined borders and contrast enhancement, measuring 27 × 14 mm (outlined with red arrows).

A follow-up thoracoabdominopelvic CT scan revealed a 5 mm subpleural granulomatous sequela in the right upper lobe, associated with a fibrotic tract. This lesion had been previously documented and remained stable in size. In the right iliac fossa, a soft tissue lesion with well-defined borders and contrast enhancement was identified, measuring 27 × 14 mm (Figure 1).

The patient underwent exploratory laparotomy with suboptimal cytoreduction, peritonectomy, and resection of multiple peritoneal implants, including a 4 cm umbilical lesion. Two implants, one involving the sigmoid colon wall and another adjacent to the left ureter, were left unresected due to high surgical risk.

Postoperative laboratory tests, as illustrated in Table 1, revealed mild anemia, slight thrombocytopenia, and an elevated C-reactive protein level, consistent with a postoperative inflammatory response. The white blood cell count remained within normal limits, and no band neutrophils were detected. Histopathological analysis confirmed that the peritoneal implants were consistent with recurrent SO.

**Table 1:** Postoperative laboratory tests. CRP: C-reactive protein; WBC: white blood cell count

Laboratory test	Result	Reference range	Interpretation
Band neutrophils	0%	0.0–4%	Normal
Hematocrit	30.5%	38.0–46.0%	Low
Hemoglobin	11.0 g/dL	12.5–15.0 g/dL	Low
WBC	7,290 uds/µL	5,000–10000 uds/µL	Normal
Platelets	139,000 uds/µL	150,000–450,000 uds/µL	Low
CRP	5.361 mg/dL	0.0–0.5 mg/dL	High

The postoperative course was uneventful. Follow-up included referrals to oncology, nutrition, and psycho-oncology, along with scheduled imaging surveillance. Systemic treatment was deferred and reserved for possible future progression of the implants.

## Discussion

As stated earlier, SO, an ovarian mature teratoma with more than 50% thyroid tissue, is rare, and its malignant transformation is extremely uncommon. In the United States, malignant struma ovarii (MSO) has an incidence of fewer than one case per 10 million annually, with approximately 10% of cases reported as malignant [1-3]. This rarity limits the number of studies and impedes the development of standardized protocols [1]. The most common histologic subtype is papillary thyroid carcinoma, as seen in our case, although follicular carcinoma is also frequently reported [2].

The disease can occur at any age, but most reported cases involve postmenopausal women, typically between 40 and 60 years of age, as in our 43-year-old patient [2,6]. Most cases of MSO are suspected based on ultrasound findings; however, other nonspecific markers, such as cancer antigen 125 (CA-125), may also be elevated, particularly because many patients remain asymptomatic [1]. However, when symptoms do occur, they are usually nonspecific, such as abdominal discomfort, vague or intermittent pelvic pain, a palpable adnexal mass, or abnormal vaginal bleeding [1,3]. In some cases, patients may present with signs of hyperthyroidism due to functional thyroid tissue within the tumor [1]. Despite the extensive pelvic disease, none of the previously described symptoms were present in our patient, and thyroid function tests remained within normal limits. The diagnosis was made during routine surveillance following the initial presentation of SO.

There are plenty of studies available for the diagnosis of SO. Imaging plays an essential yet often inconclusive role in diagnosis. On transvaginal ultrasound (TVUS), as previously mentioned, SO typically appears as a complex adnexal mass with both cystic and solid components. In some cases, the echogenicity may mimic mature cystic teratomas due to the presence of fat or hair [7]. Integration of CA-125 and human epididymis protein 4 with TVUS can improve differentiation between malignant and benign masses and can support suspicions when biomarker levels are elevated [8]. MRI offers enhanced tissue characterization, with SO presenting as a multilocular mass containing both cystic and solid areas. “Struma pearls” are hyperechoic, well-defined solid components seen within SO on ultrasound, resembling thyroid tissue. While their presence, along with comet-tail artifacts, can suggest a benign multilocular cystic lesion, these features are not specific and do not reliably exclude malignancy, especially when accompanied by suspicious findings such as irregular septations, increased vascularity, or infiltrative growth [9]. Despite these features, differentiation from ovarian carcinoma, endometrioma, or other benign neoplasms remains difficult preoperatively [7].

Other studies to consider for patients with hormonally active SO are nuclear and hybrid imaging methods. A recent systematic review found that 18F-fluorodeoxyglucose (FDG) positron emission tomography (PET)/computed tomography and radioactive iodine-131 sodium iodide single-photon emission computed tomography/computed tomography each played roles in approximately 50% of malignant cases, and their combined use improved localization and staging accuracy [10]. Another case report described a precise preoperative diagnosis using iodine-131 scintigraphy plus FDG PET in SO presenting as pseudo Meigs’ syndrome [11].

The gold standard for diagnosis rests on histopathology, which shows gelatinous, colloid-filled follicles, lined by thyroid-like epithelium and positive immunostaining for thyroglobulin and thyroid transcription factor 1, confirming the thyroid origin [7,10].

MSO presents its diagnostic challenges, mainly because its histology can mimic normal thyroid tissue. Two reported cases of highly differentiated follicular carcinoma of the ovary emerged years after initial benign diagnosis (6-14 years later), emphasizing the importance of long-term surveillance with thyroglobulin levels and potential thyroidectomy followed by RAI treatment [12]. A recent BMC case series also highlighted presentation, imaging, and treatment patterns, including salpingo-oophorectomy and RAI therapy, in malignant cases [13].

Differentiating metastatic thyroid carcinoma and primary malignant transformation of SO can be misleading, particularly in patients with a history of thyroid carcinoma. While pelvic thyroid implants may suggest metastasis, true ovarian spread is extremely rare, supporting the idea that MSO and thyroid cancer are typically independent. Although no definitive connection has been established, various studies have reported synchronous primary thyroid carcinoma. Importantly, as stated by Leuștean et al., the risk of coexistence of thyroid cancer in the neck seems significantly increased in patients with MSO compared with the general population. In our case, a history of synchronous papillary thyroid carcinoma alongside recurrent SO suggests they are independent malignancies. These findings suggest that routine thyroid imaging should be recommended in patients with MSO [1].

Recent advances in ultrasound artificial intelligence (AI)/computer-aided diagnosis technology show promise as diagnostic tools for SO/MSO. A hybrid AI pipeline analyzing echogenic components on ultrasound achieved an area under the curve of 0.93 for differentiating benign vs. malignant adnexal masses, which may help flag suspicious cases such as SO for further imaging [14].

Regarding management and treatment of SO, benign struma ovarii is typically managed with conservative surgery such as unilateral salpingo-oophorectomy or cystectomy, especially in women of reproductive age. These patients generally do not require additional interventions, and follow-ups may be done with periodic pelvic imaging, as the risk of recurrence is very low [15].

In contrast, MSO requires a more aggressive and multidisciplinary approach. Initial treatment involves surgical cytoreduction, often including total abdominal hysterectomy with bilateral salpingo-oophorectomy, especially in postmenopausal women or those not preserving fertility [13,16]. Additionally, total thyroidectomy is recommended in MSO, not only to rule out primary thyroid carcinoma but also to facilitate RAI therapy and enable thyroglobulin to serve as a reliable tumor marker for surveillance [12,17]. Recent literature recommends RAI following total thyroidectomy in cases with metastatic spread, incomplete surgical resection, or recurrence. Systemic chemotherapy, such as a platinum-taxane regimen, may be reserved for rare instances of aggressive, recurrent, or unresectable disease; although data supporting its efficacy remain limited [13]. Long-term surveillance with thyroglobulin levels and periodic imaging is essential in MSO due to its higher recurrence risk, which may manifest years after initial treatment [12,17]. Thyroglobulin levels may remain undetectable even in recurrent peritoneal disease, which highlights the limitations of relying solely on biochemical surveillance [13,15].

Our patient’s history aligns with these recommendations, as she underwent a thyroidectomy and received RAI after initial MSO diagnosis, had a favorable biochemical response, and later developed biochemically silent peritoneal recurrence. Suboptimal cytoreduction was performed to minimize morbidity, given the patient’s asymptomatic status, stability of disease on imaging, and absence of biochemical markers. As residual implants remain limited and stable, systemic therapy is withheld pending further progression. This individualized and conservative approach is consistent with consensus recommendations emphasizing clinical context, disease burden, and patient preferences [13,16].

## Conclusions

This case highlights the rarity and diagnostic complexity of MSO, particularly when it presents as a recurrence with peritoneal implants years after initial treatment. Despite its indolent course, MSO requires individualized, multidisciplinary management due to the lack of standardized protocols. Surgery remains the cornerstone of treatment, with total thyroidectomy and RAI therapy aiding diagnosis, surveillance, and disease control. We recommend long-term follow-up with periodic imaging and thyroglobulin monitoring, although the latter may be unreliable in some recurrent cases. Treatment decisions should consider disease extent, patient symptoms, and recurrence risk. In asymptomatic stable patients with limited disease, conservative management with close surveillance may be appropriate. Further research is needed to guide systemic therapy and refine follow-up strategies.
